# Evaluation of different cooling management strategies for lactating Holstein × Gir dairy cows

**DOI:** 10.1093/tas/txab199

**Published:** 2021-10-14

**Authors:** João Paulo de A Lourenço, Bruno I Cappellozza, Rafaela D Bertin, Victor F B Miranda, Wilson M C Junior, Osvaldo A de Sousa, José Luiz M Vasconcelos

**Affiliations:** 1 School of Veterinary Medicine and Animal Science, São Paulo State University (UNESP), Botucatu, SP, Brazil; 2 Nutricorp, Araras, SP, Brazil

**Keywords:** cooling management, dairy cows, heat stress, temperature-humidity index, vaginal temperature

## Abstract

Heat stress negatively impacts production, reproduction, and health of ruminants and strategies to alleviate these losses are warranted. Therefore, four experiments evaluated different cooling strategies on vaginal temperature (VT) of Holstein × Gir cows. Experiment 1 compared different amounts of water (2- or 4-L) over a 1-hour period from 1000 to 1100 h and 1600 to 1700 h. Experiment 2 evaluated the effects of sprinkling duration (in hours; 1- or 2-H), whereas Experiment 3 evaluated the effects of water amount (4- or 8-L) applied for 1- or 2-H. Lastly, the effects of a cooling strategy on specific hours of the day, starting at either 0700 (T-1) or 1100 h (T-2; Experiment 4), were evaluated. In all experiments, 12 Holstein × Gir cows were used in a 2 × 2 Latin Square Design containing two periods of 6 days each. Temperature and humidity index (THI) were recorded hourly and VT was recorded every 10-min throughout the experiments. As expected, an hour effect was observed for THI (*P* < 0.0001), which peaked early in the afternoon. In Experiment 1, a treatment × hour interaction was observed (*P* < 0.0001) for VT, as animals assigned to receive 4-L had a reduced VT at 1100, 1600, 1700, and 2300 h (*P* ≤ 0.03). During the cooling applications, cows receiving 4-L for 1 h had a reduced VT from 60 to 150 min (*P* ≤ 0.04). In Experiment 2, a treatment × hour interaction was observed (*P* < 0.0001) for VT, as animals assigned to receive 4-L of water for 2-H had a reduced VT at 1200 h (*P* = 0.05). Moreover, during the cooling process, VT was reduced for 2-H cows from 140 to 170 min after the beginning of the cooling process (*P* ≤ 0.05). In Experiment 3, animals assigned to receive 4-L + 2H had a reduced VT at 1200, 1700, 1800, and 1900 h (*P* < 0.001). A treatment × hour interaction was observed (*P* < 0.0001), as VT was reduced for 4-L + 2-H cows from 130 to 180 min after the beginning of the cooling process (*P* ≤ 0.05). In Experiment 4, by the time when the first cooling cycle of T-1 was applied (0700 h), T-1 cows consistently had (*P* ≤ 0.05) a reduced VT up to the hottest hours and greatest THI of the day (1400 and 1500 h). This pattern was maintained until the end of the last cooling cycle, whereas T-2 cows had a reduced VT. In summary, 4 L of water over a 5-min cycle for a period of 2 hours twice a day maintained VT of Holstein × Gir cows at lower levels. Moreover, the hour at which the first cooling cycle starts also should be considered when evaluating the efficacy of a cooling strategy for an entire day.

## INTRODUCTION

Heat stress is a common issue faced by dairy producers around the globe, resulting in significant impairments in metabolism (Rhoads et al., 2010; Gao et al., 2017), productive (West, 2003), reproductive (Hansen, 2009; Pereira et al., 2021), and health (do Amaral et al., 2009; Leiva et al., 2017) function of the dairy cattle herd. Additionally, recent articles demonstrated that the occurrence of heat stress in pregnant dams also impact health and productive parameters of the offspring at birth (Tao et al., 2012), 30 days post-birth (Strong et al., 2015), and later in life (Tao et al., 2019), indicating that heat stress also have transgenerational effects. Therefore, facilities, management, and nutritional strategies that reduce the occurrence of heat stress in dairy cows are warranted (Berman, 2019) to improve health, production, reproduction, and welfare of the herd.

The use of cooling strategies and fans to reduce the negative effects of heat stress, while improving health and performance of the dairy cattle herd have been given full consideration (West, 2003). To the best of our knowledge, others have evaluated cooling strategies throughout the hottest month in a specific region (Ortiz et al., 2015), whereas no other research group evaluated the effects of predetermined amounts of water, duration, and moments of the day in which cooling management strategies are used. This fact becomes even more important if we consider the amount of water and electricity often used in modern dairy production systems (Ortiz et al., 2015). Hence, we hypothesized that cooling could be more effective by employing predetermined amounts of water and/or by choosing certain hours of the day to regulate cow’s temperature. Therefore, our objective was to evaluate the efficacy of different cooling strategies, regarding amounts of water used, duration, and moment of the day in which systems are activated, on vaginal temperature (VT) of lactating multiparous Holstein × Gir dairy cows.

## MATERIALS AND METHODS

All experiments were conducted at the São Paulo State University—Lageado Experimental Station, located in Botucatu, São Paulo, Brazil (22°53′25″ S, 48°27 ′19″ W, and an elevation of 828 m). All animals utilized herein were cared for in accordance with acceptable practices and experimental protocols reviewed and approved by the São Paulo State University Institutional Animal Care and Use Committee (IACUC # 003/2017).

In all experiments (*n* = 4) that will be reported herein, animals were maintained in a single drylot pen with ad libitum access to water and a total mixed ration (TMR; 1.5 m of linear bunk space/cow). The TMR was formulated with the NRC (2001) and consisted (dry matter [DM] basis) of 60% corn silage and 40% corn-soybean meal-based concentrate containing a mineral-vitamin mix. Throughout the experimental periods, TMR was offered twice a day (0700 and 1800 h) after milking, whereas cows were also milked twice a day (0600 and 1700 h).

The facility used for all experiments consisted of one rubber-floored unshaded paddocks with 12 individual self-locking head gates (1.1 m × 1.0 m per animal). During the period animals were receiving the cooling treatments, 0.5 kg of the corn-soybean meal-based supplement was individually offered in all experiments. Moreover, in all experiments, the cooling process was performed in predetermined sprinkling cycles of 1 min with constant ventilation. Specifically for the ventilation, the fan (GEA-Magnum 52; 1 HP motor with a 54 m^3^/W, 460 rpm, and 132 cm helix; GEA Farm Technologies do Brasil, Jaguariúna, SP, Brazil) was placed 2.5 m above the floor and leaning down on the self-locking head gates, removing the air from the paddock. Sprinklers (Turbo FloodJet, Spraying Systems Co., Wheaton, IL) were placed 1.5 m above the self-locking head gates, ensuring that the back and the lateral of each animal was dripped, whereas the spray radius was 2 m with a water pressure regulator of 207 kPa (30 psi; 2.1 kg/cm^3^) and each sprinkler had a total water flow rate of 4 L/min, which was manually maintained at a steady rate over the experiments described below following the procedures described by Chen et al. (2015). Regardless of the experiment, animals were assigned to each treatment for a 6-day period and crossed-over to the other treatment for another 6 days, resulting in a 2 × 2 Latin Square Design arrangement. Considering the nature and objective of the present manuscript, all experiments reported below were conducted during the warm season in Brazil (December to March) and no artificial intervention or strategy was used to induce heat stress.

### Experiment 1

This experiment was conducted in March 2016 and 12 lactating multiparous Holstein × Gir cows (initial body weight [BW] 618 ± 72 kg, initial body condition score [BCS] 3.04 ± 0.10 (Wildman et al., 1982), days in milk 196 ± 9 days, and mean milk production 17.2 ± 2.7 kg) were used. Cows were ranked according to BCS and milk production, and then assigned to receive 2 (2-L) or 4 (4-L) liters of water per 5-min cycle over 1 h from 1000 to 1100 h and from 1600 to 1700 h (total water used per hour was 24 and 48 L for 2-L and 4-L, respectively). More specifically, treatments were applied over a 5-min cooling cycle, in which sprinklers were on for 0.5 or 1 min and off for 4.5 and 4 min, for 2-L and 4-L, respectively.

### Experiment 2

This experiment was conducted in December 2016 and 12 lactating multiparous Holstein × Gir cows (initial BW 589 ± 60 kg, initial BCS 3.13 ± 0.20, days in milk 167 ± 7 days, and mean milk production 21.9 ± 6.7 kg) were used. Cows were ranked according to BCS and milk production, and then assigned to receive 4 L of water either over a 1- (1-H) or a 2-hour (2-H) period. The 1-H treatment was applied from 1000 to 1100 h and from 1500 to 1600 h, whereas the 2-H was applied from 1000 to 1200 h and from 1500 to 1700 h. Similarly to what was reported in Experiment 1, treatments were applied over a 5-min cooling cycle, in which sprinklers were on for 1 min and off for 4 min. Additionally, after cooling cycle ended for 1-H, cows were moved to the drylot pen, where they were maintained until the subsequent cooling management.

### Experiment 3

This experiment was conducted in January 2017 and 12 lactating multiparous Holstein × Gir cows (initial BW 600 ± 71 kg, initial BCS 3.17 ± 0.25, days in milk 203 ± 8 days, and mean milk production 15.0 ± 5.0 kg) were used. Cows ranked according to BCS and milk production, and then assigned to receive 4 L of water per 5-min cycle over a 2-hour (4-L + 2-H) period or 8 L of water per 5-min cycle over a 1-hour period (8-L + 1-H). The 4-L + 2-H treatment was applied from 1000 to 1200 h and from 1500 to 1700 h, whereas the 8-L + 1-H was applied from 1000 to 1100 h and from 1500 to 1600 h. Similarly to other experiments, treatments were applied over a 5-min cooling cycle, in which sprinklers were on for 1 or 2 min and off for 4 and 3 min, for 4-L + 2-H and 8-L + 1-H, respectively. Cows assigned to both treatments received the same amount of water over the cooling period (96 L in total). Additionally, after cooling cycle ended for 8-L + 1-H, cows were moved to the drylot pen, where they were maintained until the subsequent cooling management.

### Experiment 4

This experiment was conducted in February 2017 and 12 lactating multiparous Holstein × Gir cows (initial BW 601 ± 76 kg, initial BCS 3.17 ± 0.25, days in milk 208 ± 9 days, and mean milk production 14.0 ± 4.1 kg) were used. Cows were ranked according to BCS and milk production, and then assigned to three cooling applications throughout the day, receiving 4 L of water per 5-min cycle over a predetermined 2-hour period either from 0700 to 0900 h, 1100 to 1300 h, and 1500 to 1700 h (T-1) or from 1100 to 1300 h, 1500 to 1700 h, and 1900 to 2100 h (T-2).

### Temperature and humidity index

In all experiments, environmental temperature (T) and relative humidity (RH; Willard et al., 2003) were recorded hourly using 2 HOBO Water Temp Pro V2 data loggers (Onset Company, Bourne, MA) throughout the experimental period (12 days). Each device was kept in the shade, with one device located adjacent to the drylot pen and the other device located in the individual feeding station facility. Data from both devices were downloaded with the HOBOware Pro-Mac/Win Data Logger software, which used the following equation to determine temperature and humidity index (THI) (Mader et al., 2006):


$$\begin{array}{l} \;THI=\left(0.8\times T\;{}^{\circ }C\right)+[(RH/100)\times \left(T\;{}^{\circ }C-14.4\right)]+46.4 \end{array}$$

### VT

In all experiments and throughout the experimental periods, cows were fitted intravaginally with a thermometer (iButton temperature loggers DS-1922L, Maxim Integrated, San Jose, CA), attached to a controlled internal drug-releasing device (CIDR, Zoetis, São Paulo, SP, Brazil) that did not contain hormones, and have been previously validated for dairy cattle (Brandão et al., 2016; Leiva et al., 2017). Cow VT was recorded every 10 min over a 24-hour period, whereas thermometers and CIDR devices were replaced every 3 days. For analysis during and after the cooling process, VT data were collected up to 120 min after the cooling process finished, whereas for Experiments 2 and 3, data were collected up to 270 min after the end of the cooling process. The initial idea herein was to take VT measurements up to two times the cooling management length (in min), but given that Experiments 2 and 3 had a 2-hour cooling length, this was not possible as it would coincide with the VT data for the afternoon cooling.

### Statistical analysis

Thermometers (iButton temperature loggers DS1922L) used herein were incubated for 48 h at 38 °C (Symphony Incubating Orbital Shaker Model 5000I, Troemner, LLC, Thorofare, NJ), and temperature was recorded hourly to ensure a proper functioning of the devices. During the experiment, cows were always inserted with the same thermometers.

All experiments were analyzed as a 2 × 2 Latin square design (LSD) using cow as the experimental unit. All data were analyzed using the MIXED procedure of SAS (version 9.4; SAS Inst. Inc., Cary, NC) and the Satterthwaite approximation to determine the denominator df for the test of fixed effects. Temperature and humidity relative index values were statistically analyzed containing hour in the model statement and day as an independent variable. For hourly VT data, model statement contained the effects of treatment, hour of the day, and the resulting interaction. For all analysis, day was included as independent variable. Animal(treatment) and period of the LSD were used as random statements. The specified term for the repeated statement was hour, whereas animal(treatment) was the subject. The compound symmetry (CS) structure was selected as it provided the smallest Akaike Information Criterion (AIC). For VT data during and after the cooling processes, the three timepoints prior to the beginning of cooling were used and analyzed as covariates, exception being Experiment 4 Hence, with the exception for Experiment 4, all VT data were covariately adjusted. The model statement contained the effects of treatment, hour of the day, and the resulting interaction, whereas day and moment of cooling (morning or afternoon) were included as independent variables. Animal(treatment) and period of the LSD were used as random statements. The specified term for the repeated statement was hour, whereas animal(treatment) was the subject. The CS structure was selected as it provided the smallest AIC. All results are reported as least square means and separated using the PDIFF. Significance was set at *P* ≤ 0.05 and tendencies were denoted if 0.05 < *P* ≤ 0.10. Results are reported according to the main effects if no interactions were significant or according to the highest-order interaction detected.

## RESULTS

### Experiment 1

**Figure 1.**
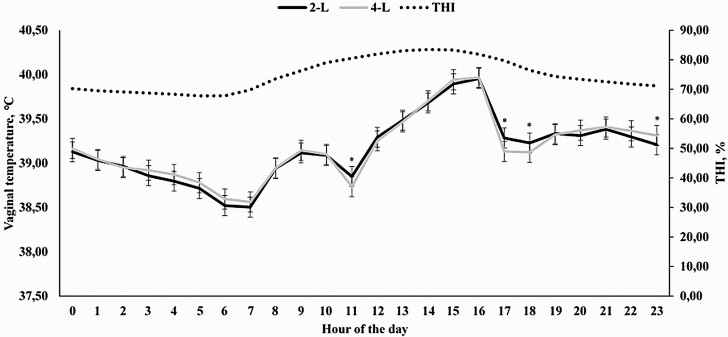
Temperature humidity index (THI) and hourly vaginal temperature of Holstein × Gir dairy cows assigned to receive 2 (2-L) or 4 liters (4-L) of water from 1000 to 1100 h and from 1600 to 1700 h in Experiment 1. An hour effect was detected for THI (*P* < 0.0001) and a treatment × hour interaction was observed for vaginal temperature (*P* < 0.01). ^*^ denotes differences at *P* ≤ 0.05.

An effect of hour of the day was observed for THI (*P* < 0.0001), which ranged from 67.85 at 0600 h to its maximum of 83.51 at 1400 h (Figure 1). As expected, a treatment × hour interaction was observed (*P* < 0.0001) for VT, as animals assigned to receive 4-L had a reduced VT at 1100, 1700, 1800, and 2300 h (*P* ≤ 0.03) when compared with cohorts assigned to receive 2-L (Figure 1).

**Figure 2.**
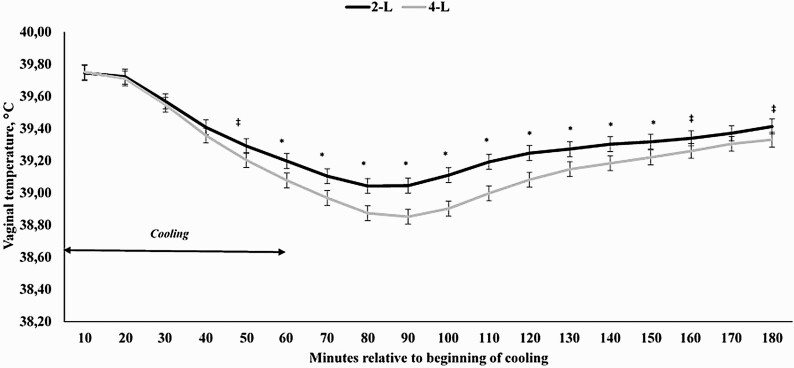
Vaginal temperature of Holstein × Gir dairy cows assigned to receive 2 (**2-L**) or 4 liters (**4-L**) of water from 1000 to 1100 h and from 1600 to 1700 h in Experiment 1. A treatment × hour interaction was observed for vaginal temperature (*P* < 0.0001). ^*^ denotes differences at *P* ≤ 0.05 and ^‡^ denotes differences at 0.05 < *P* ≤ 0.10.

When analyzing the specific period of time in which water was sprayed to the cows, a treatment × hour interaction was also detected (*P* < 0.0001; Figure 2). The covariates obtained prior to the beginning of the cooling process (three-time points) were significant (*P* < 0.0001), but did not differ among treatments (*P* = 0.17; 39.59 and 39.64 °C for 2-L and 4-L, respectively; SEM = 0.105). Cows receiving 4-L for 1 h had a reduced VT from 60 to 150 min (*P* ≤ 0.04), but also tended to have a reduced VT 50, 160, and 180 min after the cooling process started (*P* ≤ 0.09; Figure 2). For cows assigned to 2-L and 4-L, VT did not return to precooling levels by the end of the data analysis (120-min after the end of the cooling process; *P* < 0.0001). Additionally, moment of cooling, either in the morning or afternoon, was also significant (*P* < 0.0001), as cooling in the afternoon reduced VT at a greater rate than in the morning (data not shown), corroborating with the THI data in Figure 1.

### Experiment 2

**Figure 3.**
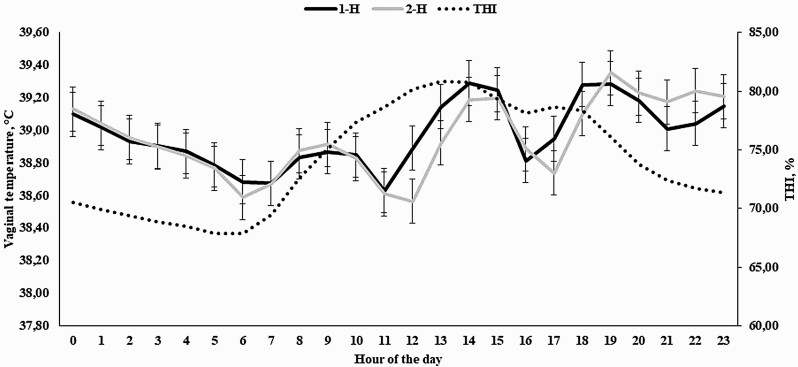
Temperature humidity index (THI) and hourly vaginal temperature of Holstein × Gir dairy cows assigned to receive 4 L of water for 1 (1-H) or 2 (2-H) hours starting at 1000 h and 1500 h in Experiment 2. An hour effect was detected for THI (*P* < 0.0001) and a treatment × hour interaction was observed for vaginal temperature (*P* < 0.0001). ^*^ denotes differences at *P* ≤ 0.05.

An hour-of-the-day effect was observed for THI (*P* < 0.0001), which ranged from 67.90 at 0500 h to its maximum of 80.81 at 1300 h (Figure 3). As expected, a treatment × hour interaction was observed (*P* < 0.0001) for VT, as animals assigned to receive 4-L of water for 2-H had a reduced VT at 1200 h (*P* = 0.05) when compared with cohorts assigned to receive the same amount of water in only 1-H (Figure 3).

**Figure 4.**
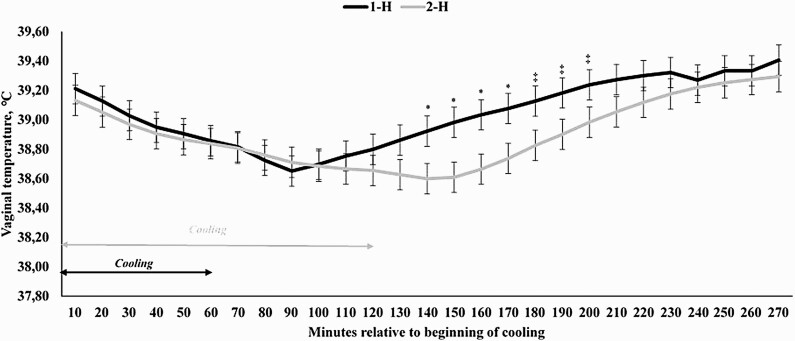
Vaginal temperature of Holstein × Gir dairy cows assigned to receive 4 L of water for 1 (1-H) or 2 (2-H) hours starting at 1000 h and 1500 h in Experiment 2. A treatment × hour interaction was observed for vaginal temperature (*P* < 0.0001). ^*^ denotes differences at *P* ≤ 0.05 and ^‡^ denotes differences at 0.05 < *P* ≤ 0.10.

The covariates analyzed prior to the beginning of the cooling process were significant (*P* < 0.0001), but did not differ between treatments (*P* = 0.85; 38.93 vs. 38.95 for 1-H and 2-H, respectively; SEM = 0.097). A treatment × hour interaction was observed (*P* < 0.0001) for VT, as VT was reduced for 2-H cows from 140 to 170 min after the beginning of the cooling process (*P* ≤ 0.04), but also tended to be reduced from 180 to 200 min (*P* ≤ 0.10; Figure 4). For cows assigned to 1-H, it took 120 min for VT to return to pre-cooling levels (*P* = 0.14), whereas for 2-H this period was of 100 min (*P* = 0.19). Lastly, the moment at which the cooling process was applied also resulted in differences on VT (*P* < 0.01), as cooling in the afternoon reduced VT at a greater rate than in the morning (data not shown), corroborating with the THI data in Figure 3.

### Experiment 3

**Figure 5.**
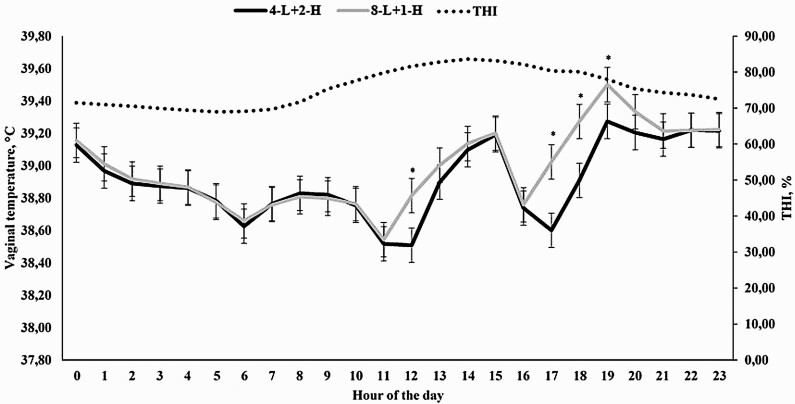
Temperature humidity index (THI) and hourly vaginal temperature of Holstein × Gir dairy cows assigned to receive either 4 L of water for 2 hours (4-L + 2-H) or 8 L of water for 1 h (8-L + 1-H) starting at 1000 h and 1500 h in Experiment 3. An hour effect was detected for THI (*P* < 0.0001) and a treatment × hour interaction was observed for vaginal temperature (*P* < 0.0001). ^*^ denotes differences at *P* ≤ 0.05 and ^‡^ denotes differences at 0.05 < *P* ≤ 0.10.

An hour-of-the-day effect was observed for THI (*P* < 0.0001), which ranged from 68.99 at 0500 h to its maximum of 83.65 at 1400 h (Figure 5). As expected, a treatment × hour interaction was observed (*P* < 0.0001) for VT, as animals assigned to receive 4-L + 2H had a reduced VT at 1200, 1700, 1800, and 1900 h (*P* < 0.001) and tended to have a reduced VT at 1300 h (*P* = 0.10) when compared with cohorts assigned to receive the same amount of water in only 1-H (8-L + 1-H; Figure 5).

**Figure 6.**
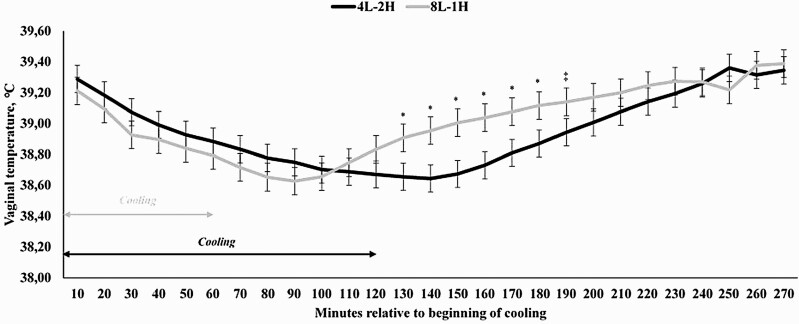
Vaginal temperature of Holstein × Gir dairy cows assigned to receive either 4 L of water for 2 h (4-L + 2-H) or 8 L of water for 1 hour (8-L + 1-H) starting at 1000 h and 1500 h in Experiment 3. A treatment × hour interaction was observed for vaginal temperature (*P* < 0.0001). ^*^ denotes differences at *P* ≤ 0.05 and ^‡^ denotes differences at 0.05 < *P* ≤ 0.10.

The covariates analyzed prior to the beginning of the cooling process were significant (*P* < 0.0001), but did not differ between treatments (*P* = 0.72; 39.07 vs. 39.08 for 4-L + 2-H and 8-L + 1-H, respectively; SEM = 0.112). A treatment × hour interaction was observed (*P* < 0.0001) for VT, as VT was reduced for 4-L + 2-H cows from 130 to 180 min after the beginning of the cooling process (*P* ≤ 0.03) and tended to be reduced at 190 min versus 8-L + 1-H (*P* = 0.08; Figure 6). For cows assigned to 4-L + 2-H, it took 110 min for VT to return to precooling levels (*P* = 0.15), whereas for 8-L + 1-H this period was of 100 min (*P* = 0.18). Moreover, the moment at which the cooling process was applied did not impact VT (*P* = 0.40).

### Experiment 4

**Figure 7.**
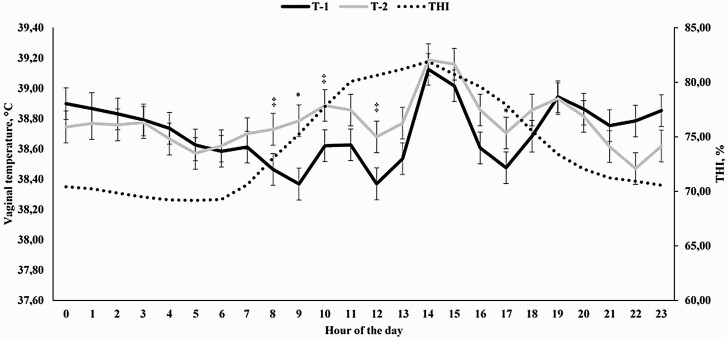
Temperature humidity index (THI) and vaginal rectal temperature of Holstein × Gir dairy cows assigned to receive either 4 L of water for 2 h starting at either 0700, 1100, and 1500 h (T-1) or at 1100, 1500, and 1900 h (T-2) in Experiment 4. An hour effect was detected for THI (*P* < 0.0001) and a treatment × hour interaction was observed for vaginal temperature (*P* < 0.0001). ^*^ denotes differences at *P* ≤ 0.05 and ^‡^ denotes differences at 0.05 < *P* ≤ 0.10.

An hour-of-the-day effect was observed for THI (*P* < 0.0001), which ranged from 69.17 at 0500 h to its maximum of 81.90 at 1400 h (Figure 7). As expected, a treatment × hour interaction was observed (*P* < 0.0001) for VT, as animals assigned to T-1 had a reduced VT at 0900 and 2200 h (*P* ≤ 0.05) and tended to have a reduced VT at 0800, 1000, and 1200 h (*P* ≤ 0.10) when compared with cohorts receiving T-2 (Figure 7).

**Figure 8.**
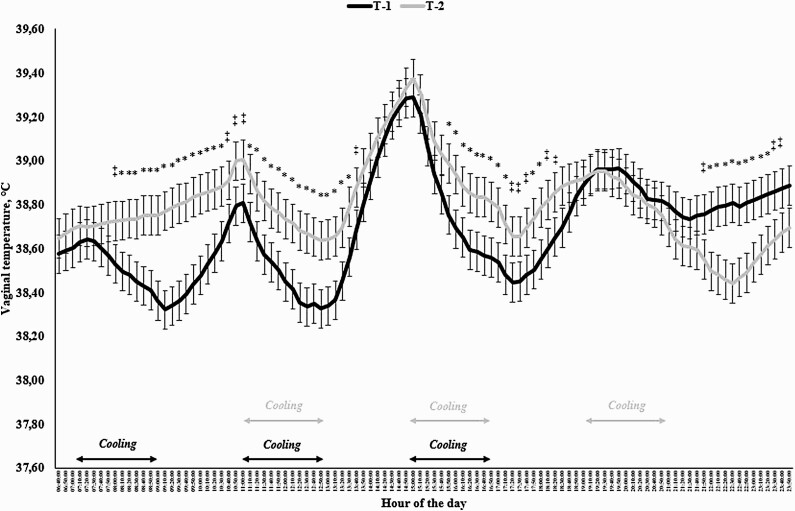
Vaginal temperature of Holstein × Gir dairy cows assigned to receive either 4 L of water for 2 h starting at either 0700, 1100, and 1500 h (T-1) or at 1100, 1500, and 1900 h (T-2) in Experiment 4. A treatment × hour interaction was observed for vaginal temperature (*P* < 0.0001). ^*^ denotes differences at *P* ≤ 0.05 and ^‡^ denotes differences at 0.05 < *P* ≤ 0.10.

By the time when the first cooling cycle of T-1 was applied (0700 h), T-1 cows consistently had (*P* ≤ 0.05) or tended to have (*P* ≤ 0.10) reduced VT versus T-2 cows (Figure 8), up to the hottest hours and greatest THI of the day (1400 and 1500 h), when VT did not differ between T-1 and T-2 cows (*P* ≥ 0.33). The pattern of reduced VT (*P* ≤ 0.10) in T-1 cows was maintained until the end of the last cooling cycle, whereas T-2 cows had a reduced VT versus T-1 cows following the end of the third cooling cycle (*P* ≤ 0.10; Figure 8).

## DISCUSSION

The primary goal of these experiments was to evaluate the effects of different cooling strategies to alleviate heat stress in lactating Holstein × Gir multiparous dairy cows. This becomes paramount on modern dairy production settings given the growing need of a more efficient water utilization in agriculture (World Resources Institute, 2011) and the fact that this natural resource might become scarce in the near future (FAO, 2009). As stated by Berman (2019), air temperature and humidity are the most difficult components to modify in a farming system environment, from both technological and cost-benefit considerations. Therefore, not only the amount of water used, but also the moment at which cooling systems are activated should be evaluated to improve the efficacy by which cow temperature is maintained below heat stress threshold and, also, the efficiency of water utilization in dairy farms.

The present experiments have not evaluated dry matter intake (DMI), milk production, and milk composition as the experimental period was considered short (6-day period in each treatment and 12-day total for each experiment) for such parameters to be analyzed. Nonetheless, the benefits of maintaining dairy cows under a lower temperature threshold have been reported by several authors evaluating different cooling strategies. Nutritional (Brandão et al., 2016; Leiva et al., 2017), genetic, and management (West, 2003) strategies have been reported to improve DMI, milk production and composition, health, metabolism, and reproductive function of dairy cows, or in other words, have demonstrated how heat stress might impair these productive and reproductive parameters in dairy cows (West, 2003; García-Ispierto et al., 2007; Roth, 2008; Van Os, 2019; Bai et al., 2020; Pereira et al., 2021). Moreover, it is well-known that heat stress occurrence in pregnant dairy cows and heifers impacts gestation length (Tao et al., 2012; Monteiro et al., 2014; Davidson et al., 2021a) and impacts fetal intrauterine development (Tao et al., 2012; Monteiro et al., 2014; Dado-Senn et al., 2020), early after birth (Strong et al., 2015; Davidson et al., 2021a), but even more importantly, later in the dairy female’s productive life (Laporta et al., 2017; Tao et al., 2019; Davidson et al., 2021b).

As expected, in all experiments, an hour effect was observed on THI values, with the peak usually occurring in the first PM hours. In fact, the % of time in which THI remained above the threshold (>68) was 91.7%, 91.7%, 100%, and 100% from Experiments 1 to 4, respectively. Recently, our research group (Leiva et al., 2017) reported similar THI values for Holstein × Gir dairy cows raised under the same environment and, highlighting the fact that animals were heat-stressed throughout the days of the experiments evaluated herein (Zimbleman et al., 2009). It is also noteworthy the fact that THI remained above 68 even in the evenings, which might pose a challenge for cooling the dairy cow herd (Berman, 2019; Van Os, 2019) and, therefore, cooling management strategies must be employed to alleviate the permanent heat stress state the herd is going through.

In Experiments 1 and 2, moment at which cooling was applied to the animals was significant, demonstrating that higher threshold values are often observed in the afternoon, when THI was often greater (Figures 1, 3, 5, and 7). Tresoldi et al. (2018) also reported significant effects on the hour in which spray was applied on temperature of dairy cows. Similarly, Leiva et al. (2017) also demonstrated greater THI values in the end of the morning and early afternoon, which affected VT of dairy cows. Therefore, the utilization of morning and afternoon cooling strategies was assertive and important for the present work. One might speculate that the specific differences on morning and afternoon VT should be evaluated, but the primary objective of the present experiment was not to compare whether morning or afternoon was more effective in a cooling process, but to demonstrate the efficacy of cooling strategies, contemplating both morning and afternoon periods, employed in different hours of the day, which would also fit greater THI values.

Results from Experiment 1 demonstrate that 4-L of water per 5-min cycle was more effective in maintaining VT of dairy cows lower for a longer period of time, a fact that becomes even more important when we consider the end of the cooling application cycle. In the present study, cows assigned to 2- and 4-L of water per min used 48 and 96 L of water/head per day, which is considerably less than previously reported by other research groups (Ortiz et al., 2015). It was not the aim of the present study to elucidate a specific amount of water to be used as a cooling strategy for modern dairy operations, but it should be taken into consideration when designing a cooling management program. As an example, other researchers have failed to demonstrate associative benefits on core body temperature and cooling when a single 12-min application of 4.8 L of water (Chen et al., 2016) or 4 repeated spray applications of 1.2 L (Chen et al., 2015) were used. In a recent research trial, Tresoldi et al. (2018) demonstrated that spraying dairy cows for a longer period of time and, consequently, receiving a greater amount of water (in L), resulted in a greater reduction in respiratory rate and skin temperatures for 30 min after the spray was turned off. Our data demonstrated that VT of dairy cows was reduced for 4-L versus 2-L cows for up to 120 min after the cooling process was finished.

In a subsequent approach, Experiment 2 evaluated the efficacy of 4 L of water applied per 5-min cycle in different cooling lengths, either for 1- or 2-H. In fact, applying the cooling strategy for 2-H was more effective in maintaining VT at lower values versus dairy cows receiving the cooling strategy for 1-H only. Recently, Tresoldi et al. (2018) also reported that longer periods of evaporative strategies were more effective in reducing respiratory rate and skin temperature of dairy cows. Edstrom (2011) stated that cows should be soaked water to the hide along the topline while not getting wet to the point water running off the sides. Additionally, Tresoldi et al. (2018) speculated that when the water is sprayed for a longer period of time, there are likely more opportunities for the heat to be transferred from the body of the cow to the dripping water when compared with shorter duration sprays. Along with the cooler microclimate that likely results when droplets evaporate from the ground in the air, dripping water generates rapid reductions in skin temperature and respiration rate (Van Os, 2019), which were indeed reduced after a spray application rate of 1.3 L/min for a 3-min cycle (Chen et al., 2015) and 4.9 L/min for a 90-sec cycle (Tresoldi et al., 2018).

Corroborating with the later rationale and in agreement with data from Experiment 3, the duration of the cooling cycle seems to be more important than the amount of water used per se. As aforementioned and in agreement with our results, Tresoldi et al. (2018) reported that longer periods of spraying reduced respiratory rate and skin temperature of dairy cows. Nonetheless, these authors have not reported positive effects of longer cooling strategies during the drying phase, which differs from our data, indicating that heat was being removed only when water was being sprayed in the cows (Tresoldi et al., 2018). Our results demonstrated that besides positive effects during the cooling period, applying 4-L + 2-H reduced VT for 70 min after cooling had finished. Tresoldi et al. (2018) reported that, regardless of whether cows are soaked only enough to wet the back or if water drips from their sides, the coat takes roughly 14 to 16 min to dry and this range might depend on the weather where dairy cows are being reared on (Van Os, 2019). As stated by Van Os (2019), it seems that the optimal volume of water to be used is approximately 4 L per spray application, considering at least 4 to 5 cycles per hour. Moreover, opposite than others might speculate, a sudden increase in the amount of water supplied to the herd might result in a pattern of diminishing returns for the cooling strategy (Van Os, 2019).

The key interesting findings from the present manuscript are the fact that (1) VT of dairy cows are maintained at a steady value for a longer period of time than previously reported when the cooling cycle ends and (2) VT values return to precooling ones might be dependent on the length of the cooling cycle. To the best of our knowledge, this is the first manuscript reporting this information when evaluating cooling strategies for lactating dairy cows. The former statement is based on the fact that even when the cooling cycle is finished, VT is not further decreased, even though the hair skin is wet. Nonetheless, it is still effective in maintaining lower minimum threshold temperature levels, which have not been previously reported by others (Ortiz et al., 2015; Tresoldi et al., 2018; Van Os, 2019). The second statement might be even more important and is related to the fact that if dairy cows are cooled for 120 min, it takes approximately the same period of time for VT to return to precooling levels (Figures 4 and 6), suggesting that the animal might have a thermoregulatory control mediating this process. Nonetheless, the presence and/or the mechanism by which this might occur is unknown and warrants further research efforts. Moreover, it is noteworthy that data from Experiment 1 were not taken into consideration for this later statement, as there might be a confounding effect with the volume of water applied in the cooling system and from the fact that during the data evaluation period, VT values did not return to precooling baseline values.

One might question the biological efficacy and/or relevance of the treatments evaluated from Experiments 1 to 3 in actually reducing heat stress, as VT often reached 39.2 °C post-cooling. Vaginal and body temperature returned to precooling values as cows were drying off the cooling cycles evaluated from 1 to 2 h, in different amounts of water. Hence, the differences on VT observed and reported herein are extremely relevant to the animals, ensuring heat stress was alleviated for a period of time, whereas the biological and productive effects of these differences should be evaluated in a further experiment.

Lastly, it is clear that the hour at which the first cooling cycle starts significantly impacts VT of dairy cows. This rationale is based on findings of Experiment 4, in which cows receiving the first cooling cycle early in the morning (0700 h) maintained a lower VT throughout the day, even when both treatments received the cooling program at the same time of the day (1100 and 1500 h; Figure 8). Nonetheless, the reverse occurred following the last cooling cycle of T-2 cows, which had a reduced VT versus cows from T-1 that received the last cooling cycle at 1500 h. To the best of our knowledge, this is the first research trial evaluating the effects of cooling on specific hours of the day versus VT of dairy cows. Ortiz et al. (2015) also evaluated the effects of two cooling systems on daily core body temperature of dairy cows during a warm month in Saudi Arabia, but both cooling systems were turned on and off at the same time, which pose a challenge in understanding the effects of hour of the day on the thermal responses. One might speculate that cows should be cooled when the THI starts to increase (Figure 7) to maintain VT at lower levels throughout the day and that cooling cycles of 2-h duration with a 4-h interval might be an interesting strategy to reduce core body temperature of dairy cows. Therefore, alleviating the increase on VT by activating the cooling system when a concomitant increase in THI is occurring could be used as a feasible alternative to be adopted by dairy producers. Moreover, additional research is warranted to evaluate the effects of these strategies on behavior, DMI, production, health, and reproduction of dairy cows exposed to heat stress situations.

In summary, the utilization of 4 L of water over a 5-min cycle for a period of 2 h twice a day maintained VT of Holstein × Gir cows at lower levels when compared with other cooling strategies, such as amount of water and/or 1-hour period. Moreover, the hour at which the first cooling cycle starts is important when considering the efficacy of a cooling strategy for an entire day. Nonetheless, more research is warranted to evaluate these cooling approaches used herein on productive and reproductive performance, as well as health and behavior of dairy animals.
